# Multimodal Biomedical Data Fusion Using Sparse Canonical Correlation Analysis and Cooperative Learning: A Cohort Study on COVID-19

**DOI:** 10.21203/rs.3.rs-3569833/v1

**Published:** 2023-11-20

**Authors:** Ahmet Gorkem Er, Daisy Yi Ding, Berrin Er, Mertcan Uzun, Mehmet Cakmak, Christoph Sadee, Gamze Durhan, Mustafa Nasuh Ozmen, Mine Durusu Tanriover, Arzu Topeli, Yesim Aydin Son, Robert Tibshirani, Serhat Unal, Olivier Gevaert

**Affiliations:** 1) Stanford Center for Biomedical Informatics Research (BMIR), Department of Medicine, Stanford University, Stanford, CA, 94305, USA; 2) Department of Health Informatics, Graduate School of Informatics, Middle East Technical University, Ankara, 06800, Türkiye; 3) Department of Infectious Diseases and Clinical Microbiology, Hacettepe University Faculty of Medicine, Ankara, 06230, Türkiye; 4) Department of Biomedical Data Science, Stanford University, Stanford, CA, 94305, USA; 5) Department of Internal Medicine, Division of Intensive Care Medicine, Hacettepe University Faculty of Medicine, Ankara, 06230, Türkiye; 6) Department of Internal Medicine, Hacettepe University Faculty of Medicine, Ankara, 06230, Türkiye; 7) Department of Radiology, Hacettepe University Faculty of Medicine, Ankara, 06230, Türkiye; 8) Department of Statistics, Stanford University, Stanford, CA, 94305, USA

## Abstract

Through technological innovations, patient cohorts can be examined from multiple views with high-dimensional, multiscale biomedical data to classify clinical phenotypes and predict outcomes. Here, we aim to present our approach for analyzing multimodal data using unsupervised and supervised sparse linear methods in a COVID-19 patient cohort. This prospective cohort study of 149 adult patients was conducted in a tertiary care academic center. First, we used sparse canonical correlation analysis (CCA) to identify and quantify relationships across different data modalities, including viral genome sequencing, imaging, clinical data, and laboratory results. Then, we used cooperative learning to predict the clinical outcome of COVID-19 patients. We show that serum biomarkers representing severe disease and acute phase response correlate with original and wavelet radiomics features in the LLL frequency channel (*corr*(*Xu*1, *Zv*1) = 0.596, p-value < 0.001). Among radiomics features, histogram-based first-order features reporting the skewness, kurtosis, and uniformity have the lowest negative, whereas entropy-related features have the highest positive coefficients. Moreover, unsupervised analysis of clinical data and laboratory results gives insights into distinct clinical phenotypes. Leveraging the availability of global viral genome databases, we demonstrate that the Word2Vec natural language processing model can be used for viral genome encoding. It not only separates major SARS-CoV-2 variants but also allows the preservation of phylogenetic relationships among them. Our quadruple model using Word2Vec encoding achieves better prediction results in the supervised task. The model yields area under the curve (AUC) and accuracy values of 0.87 and 0.77, respectively. Our study illustrates that sparse CCA analysis and cooperative learning are powerful techniques for handling high-dimensional, multimodal data to investigate multivariate associations in unsupervised and supervised tasks.

## Introduction

In recent years, the power of multi-modal data fusion in biomedical research has become increasingly evident. Technological innovations allow us to study a patient or a cohort from multiple perspectives using high-dimensional, multiscale biomedical data. Examples of such biomedical data include clinical (electronic health records, clinical notes, laboratory results), pathological (histopathology examinations, immunofluorescence staining), molecular (DNA and RNA sequences, transcriptomics, epigenetics), and imaging (X-ray, CT, MRI) data. Machine learning methodologies have been pivotal in combining and analyzing these data modalities, unveiling a myriad of biomarkers that can be harnessed for personalized medicine applications ^1–5^. Pioneer studies in multi-modal data fusion have mainly been developed in oncology: advances in next-generation sequencing, transitioning from conventional histopathology to whole slide imaging, the comprehensive usage of radiological images, and establishing standardized, publicly available large datasets, such as The Cancer Genome Atlas (TCGA), has been a significant catalyst for these studies ^2,6^.

Coronavirus Disease 2019 (COVID-19), caused by the SARS-CoV-2 virus, was declared a pandemic by the World Health Organization (WHO) on March 11, 2020, impacting millions worldwide. As of June 10, 2023, nearly 750 million confirmed cases, including 7 million deaths, were diagnosed, with actual numbers speculated to be much higher ^7,8^. The clinical trajectory of COVID-19 patients varies significantly, and addressing this variation becomes crucial in patient management. Factors like male sex, age, and comorbidities have been tied to disease severity ^9,10^. Anomalies in laboratory results, radiological abnormalities, and the presence of specific viral mutations can also influence the clinical course of the disease ^11–18^. A plethora of clinical, laboratory, imaging, and viral genome sequencing data is available for research, and COVID-19 offers a promising avenue for applying multi-modal data fusion. However, despite the potential of multi-modal data fusion, its application to certain global health crises, like the COVID-19 pandemic, presents unique challenges. Notably, while databases for viral genome sequencing, such as GISAID and NCBI, are available, a consolidated approach to link diverse datasets, including imaging and clinical information, remains challenging ^19–21^.

This paper presents our approach for analyzing multi-modal data in a COVID-19 patient cohort using unsupervised and supervised sparse linear methods. More specifically, we use canonical correlation analysis (CCA) and cooperative learning to understand the relationships between various data modalities and predict intensive care unit (ICU) admission, respectively ^22–24^.

## Materials and Methods

### Study design and data collection

This prospective cohort study was conducted in a tertiary care academic center in Ankara, Turkey, between December 22, 2020, and May 5, 2021. The cohort consisted of two groups of patients: one group recruited as a part of the project entitled “Viral Genome Analysis in COVID-19 Patients and Genotyping of Genetic Variants Shown in the Literature Related to the Severe Course of the Disease in Humans” and the other group recruited within the scope of the Global Influenza Hospital Surveillance Network Project (GIHSN)-2020-21 in Turkey ^25^. Ethical approvals were obtained from the Institutional Ethics Committee with the code numbers GO 2021/02-22, GO 20-102, and GO 22-1211. Good clinical and laboratory practices were followed throughout the study.

Adult patients (≥18 years of age) hospitalized in the medical wards or intensive care units (ICU) who were positive for SARS-CoV-2 polymerase chain reaction (PCR) test within the last 120 hours and gave informed consent were included in the study. COVID-19 patients with at least one of the below criteria were admitted to the ICU:

- Dyspnea and respiratory distress,
- Respiratory rate >30/min,
- PaO_2_/FiO_2_ < 300 mmHg,
- Increased oxygen demand during follow-up,
- SpO_2_ <90% or PO_2_ <70 mmHg despite 5L/min O_2_ support,
- Hypotension (Systolic blood pressure <90 mmHg or more than 40 mmHg drop from usual systolic blood pressure level or mean arterial blood pressure <65 mmHg),
- Development of acute organ dysfunction such as acute kidney injury, acute elevation in liver function tests, confusion, acute bleeding diathesis,
- Elevated serum troponin with arrhythmia,
- Lactate >2 mmol/L

Relevant clinical information was gathered through face-to-face interviews with patients and attending physicians and by reviewing clinical records. Age, sex, comorbidities such as diabetes, heart failure, coronary artery disease, hypertension, malignancy, polymerase chain reaction (PCR) test results, vaccination history, medications and therapies, outcomes, laboratory results, GISAID EPI_SET identifiers, and imaging data were recorded. Since laboratory results can change within days or even hours in COVID-19 patients, only results at the time of imaging were included. All the information related to patients has been anonymized.

### Sampling and next-generation viral genome sequencing protocol

A nasopharyngeal swab or nasal specimen combined with an oropharyngeal swab was obtained from conscious patients, and a tracheal aspirate from intubated patients, in case they comply with inclusion criteria. Medical Wire M40-A Compliant Sigma-Virocult^™^ Viral Collection and Transport System combining open-bud Sigma-Swab^™^ with Virocult^™^ medium was used. EZ1 Virus mini kit V2.0 (Catalog number: 955134, Qiagen, Germany) was used for total nucleic acid extraction. Samples were directly introduced to the sequencing platform after nucleic acid extraction. Library preparation was performed using Respiratory Virus Oligos Panel V2 (Illumina Inc., #20044311) and Illumina RNA Prep with Enrichment, (L) Tagmentation (Illumina Inc., #20040537) kits according to official protocol. Before sequencing, the libraries were quantified and checked on Qubit 4 Fluorometer (ThermoFisher Inc.) and 2100 Bioanalyzer systems (Agilent Inc.). The sequencing was performed on Illumina NextSeq 550 platforms with 1.000.000 reads (2×150 bp) per sample on average. Quality control of the raw data was analyzed using the FASTQC tool. The quality passed samples were uploaded to BaseSpace (Illumina Inc.) for bioinformatics analysis. The data were analyzed using the DRAGEN COVID Lineage app (v.3.5.4) on the BaseSpace platform. The low-quality and low-variant fraction variants were filtered out (coverage > 10). The filtered variants were submitted to the GISAID database^19^.

### Identifying viral mutations and construction of phylogenetic tree

Viral genome sequences were retrieved from the GISAID database (Supplementary Table 1). Nextclade CLI (v.2.12.0) was used for sequence alignment and identifying the nucleotide and amino acid mutations and variant clades ^26^. The reference genome was determined as SARS-CoV-2 isolate Wuhan-Hu-1, GenBank: MN908947.3. Isolated study strains’ phylogenetic tree construction was performed using an optimized substitution model (GTR+F+I) according to the lowest Bayesian Information Criterion (BIC) score obtained by the ModelFinder approach and followed by ultrafast bootstrap analysis (175 iterations) on the IQ-TREE software (v.1.6.12) ^27–29^. Consensus tree annotation and visualization were then completed using the ggtree (v.3.8.0) R package ^30^.

### Viral feature preprocessing

Before utilizing any machine learning model on a genome or amino acid sequence, it is necessary to convert the sequence into a numerical format of fixed length to construct an embedding space. For this purpose, various techniques available in the literature are broadly classified as alignment-free and alignment-based methods ^31^. Alignment-free methods are mainly divided into word-based and information theory-based methods. While word-based methods rely on discovering the frequency of words (k-mers) within sequences and use similarity or dissimilarity measures derived from these patterns, information theory-based methods capture the information shared among sequences using entropy or complexity metrics ^32^. On the other hand, even though alignment-based methods have some disadvantages, such as becoming computationally expensive or assuming that homologous sequences share conserved sequences, they still constitute a well-established approach in phylogenetic studies. During the COVID-19 pandemic, Pango, Nextclade, and WHO classification systems have been widely used, and all these systems essentially rely on this methodology ^33,34^. Also, they were used in machine-learning prediction studies for creating viral feature embeddings ^35,36^.

We tried and compared two different techniques for viral encoding.

- Viral-Binary encoding: As a typical example of alignment-based methods, according to whether mutations were present or were not, each mutation was encoded as “0” or “1”. 439 unique amino acid mutations were identified in 105 isolated study strains. This created a binary column for each mutation and returned a sparse matrix.
- Viral-Word2Vec encoding: Since well-established viral genome databases exist on a global scale, we aimed to combine alignment-free and alignment-based models leveraging the Word2Vec natural language processing (NLP) model to reduce the size of the embedding space and extract the semantic relationship between each of the mutations and the strains themselves ^37,38^. We treated amino acid mutations as words and strains as sentences and used the Skip-Gram model architecture that predicts surrounding mutations in a context window given the current mutation. To construct the corpus, viral strains whose outcomes were known and collected between December 30, 2019, and March 2, 2023, on the GISAID database were used. After collecting the data, we defined the outlier strains as falling below Q1 − 1.5 IQR or above Q3 + 1.5 IQR in terms of the number of mutations. As a result, 653,134 viral genomes were used for constructing the corpus. The maximum number of mutations per strain was found to be 115, and accordingly, this number was chosen as the context window to train the Word2Vec model with vector dimension 300. The vocabulary size (the number of unique amino acid mutations) was found to be 52,311. Strain embeddings were calculated by getting the strains’ mean vector of amino acid mutations. Multi-dimensional scaling (MDS) was used to reduce dimensionality and visualize 2D plots ^39^. Cosine similarities between the strains were computed to construct a dissimilarity matrix, and this matrix was used as the precomputed dissimilarity metric in MDS.

### Imaging Data Collection and Analysis

For each patient with longitudinal CT images, the images were selected based on the following criteria: If the patient had no history of ICU admission, we included the last image at admission, as the lesions would become more apparent. However, if there was a history of ICU admission, we included the image closest to the date of ICU admission since superinfections during the ICU stay could mask lesions that might be characteristic of COVID-19. Image data were obtained from SIEMENS scanners. CT section thickness was as follows: less than 1 mm (7 patients, 5.5%), greater than or equal to 1, less than 2 mm (119 patients, 93.7%), and greater than or equal to 2 mm, less than 3 mm (1 patient, 0.01%). All images were visualized in 3D Slicer (v.5.3.0), and the right and left lungs were segmented with U-net-based pre-trained lungmask R231^40,41^. 18 first-order, 14 shape, 22 Gray-level co-occurrence matrix (GLCM), 16 Gray-level size zone matrix (GLSZM), and 14 Gray-level dependence matrix (GLDM) features were extracted from original and wavelet-filtered images from each lung using pyradiomics (v.3.0.1)^42^. All images were normalized with a scale of 500. ‘sitkBSpline’ was used as the interpolator for resampling pixel space to (1.0,1.0,1.0) mm. A fixed bin number of 64 was used for all analyses.

### Data Fusion Methodologies

#### Sparse Canonical Correlation Analysis

Assume we have two data matrices, X and Z, of dimensions n×p and n×q on the same set of n observations,

$$X=\left[\begin{array}{cccc} x_{11} & x_{12} & \cdots & x_{1p}\\ x_{21} & x_{22} & \cdots & x_{2p}\\ \vdots & \vdots & \ddots & \vdots \\ x_{n1} & x_{n2} & \cdots & x_{np} \end{array}\right]\;Z=\left[\begin{array}{cccc} z_{11} & z_{12} & \cdots & z_{1q}\\ z_{21} & z_{22} & \cdots & z_{2q}\\ \vdots & \vdots & \ddots & \vdots \\ z_{n1} & z_{n2} & \cdots & z_{nq} \end{array}\right]$$

Canonical correlation analysis (CCA) seeks linear combinations (canonical variables) of the variables in X and Z that are maximally correlated. That is, $u_{1}={\left(u_{11},u_{21},\ldots ,u_{p1}\right)}^{T}$ and $v_{1}=\left(v_{11},v_{21},\right.{\left.\ldots ,v_{q1}\right)}^{T}$ maximize $corr\left(Xu_{1},Zv_{1}\right)$. Here, we refer to $u_{1}$ and $v_{1}$ as the canonical vectors and $Xu_{1}$ and $Zv_{1}$ as the canonical variables. u and v have the dimensions of p × K and q × K for K canonical vectors, which are not correlated with each other.

Sparse CCA aims to find sparse canonical vectors u and v *such that* $u^{T}X^{T}Zv$ is optimized. The analysis was conducted using the PMA (v.1.2.1) package ^23^, where we used the CCA.permute function for selecting the sparsity parameters. Multi-CCA was used as an extension where we assessed the relationship between more than two data modalities. The p-values were calculated through permutation with the MultiCCA.permute function.

#### Cooperative Learning

Cooperative learning was used for the supervised learning task to build prediction models for the outcome of ICU hospitalization with multimodal data, which uses an agreement penalty to encourage alignment between predictions from different data modalities. By varying the weight of the agreement penalty, it provides a continuum of solutions that include the early and late fusion approaches. Using cross-validation (CV), we chose the degree of agreement, i.e., the optimal weight on the agreement penalty. Cooperative learning also combines the lasso penalty with the agreement penalty, yielding feature sparsity. Analyses were performed with the multiview (v.0.8) package ^24^.

We used a repeated stratified nested CV framework for hyperparameter tuning, model selection, and assessment (Supplementary Figure 1). 10-fold CV was performed, with the loss function as “deviance” for tuning the elastic-net mixing parameter and the weight of the agreement penalty in the inner loop. The outer loop assessed the performance of models trained in the inner loop. The final performance scores were averaged after a 5-fold CV. We conducted each experiment 30 times.

### Statistical Analysis

Descriptive statistics were used to calculate frequency and percent distributions. After testing assumptions of normality, the mean and standard deviation were used for continuous variables with normal distribution and the median and interquartile range for continuous variables without normal distribution. The statistical significance of the differences between groups was tested using Chi-square and Fischer’s exact Chi-square tests for categorical variables, an independent two-sided t-test for continuous variables with normal distribution, and a Mann-Whitney U test for continuous variables without normal distribution. The equality of variances of the results of cooperative learning was assessed with Bartlett’s test. If the hypothesis of equal variances was rejected, Welch’s ANOVA was used to test the significance between three or more groups. The Games-Howell post hoc test was used as the nonparametric approach to compare pairwise results of cooperative learning. Type I error was set at 0.05 for all analyses.

## Results

### Descriptive analysis

In total, 149 patients were enrolled in the study, and 63 patients (42.3%) were admitted to the ICU (Table 1). The mean age was 57.6±16.2 years, and 61 patients (40.9%) were female. Hypertension (48.3%), diabetes mellitus (29.5%), and coronary artery disease (22.1%) were the most common comorbidities. The mean age was higher in the ICU group compared to the non-ICU group (63.9±15.0 vs. 53.1±15.6, p<0.001). The median age-adjusted Charlson comorbidity index (CCI) was 2 (1–3) in patients hospitalized in ICU compared to 1 (0–1) in those not hospitalized in ICU (p<0.001). Age, sex, comorbidities, and CCI were used as clinical variables in downstream tasks.

**Fig. 1:**
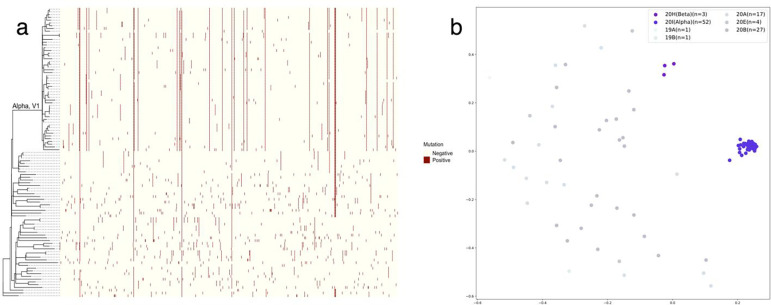
Phylogenetic tree, nucleotide substitution matrix, and Word2Vec encoding plot of isolated SARS-CoV-2 strains. **a** The phylogenetic tree of isolated SARS-CoV-2 strains and nucleotide substitutions in matrix form, in which the presence of substitutions is shown in dark red. **b** The Word2Vec encoding plot of the same strains. The nucleotide substitution matrix and Word2Vec encoding plot represent that Alpha strains appear more similar compared to non-Alpha strains.

The number of patients who underwent chest CT at least once was 127 (Supplementary Figure 2). In 105 isolated viral genome samples, the number of unique nucleotide mutations was 710, and the number of unique amino acid mutations was 439. The median of nucleotide mutations per strain was 29.0 (21.0–33.0), and the median of amino acid mutations was 22.5 (12.0–27.8) (Supplementary Figure 3). Fifty-two strains (49.5%) were assigned to Variant 20I (Alpha, V1) according to the Nextclade clades (Figure 1).

### Visualization of the global SARS-CoV-2 strains using Word2Vec embedding

**Fig 2:**
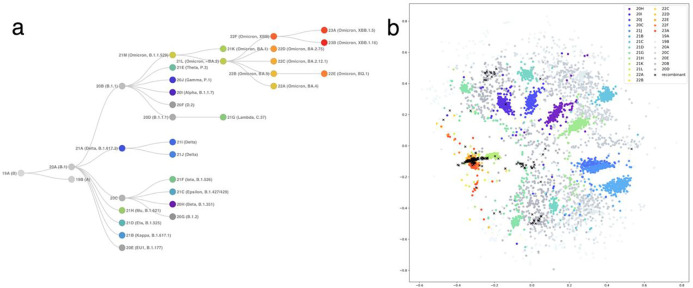
Phylogenetic tree and 2D Word2Vec encoding plot of global SARS-CoV-2 strains. **a** The phylogenetic relationships of the global SARS-CoV-2 clades as defined by Nextstrain. The screenshot was taken from CoVariants.org ^44^. **b** the Word2Vec encoding plot of 300 randomly selected viral strains from each Nextclade clade. Major variants, such as Variants 20I (Alpha, V1), 20H (Beta, V2), 21I, and 21J (Delta’s), and Omicron clades, are successfully separated.

We visualized 300 randomly selected viral strains from each Nextclade clade in the corpus, generated with global viral genome sequences on the GISAID database. This shows that major variants, such as Variants 20I (Alpha, V1), 20H (Beta, V2), 21I, and 21J (Delta’s), and Omicron clades, were successfully separated (Figure 2). Not only separation but also some of the characteristic features seen in phylogenetic relationships were observed in the embedding space. While more ancestral clades, for instance, Variants 19A, 19B, and 20A, representing the early days of the pandemic, had a wider distribution, clades dating to later periods tend to be observed within clusters. Furthermore, clades that have closer evolutionary relationships, for example, Variants 20H (Beta, V2), 21I (Delta), and 21H (Mu) were located closer in the embedding space. On the other hand, Omicron variants were separated from these groups, as highlighted in the literature ^43^. It was also seen that some recombinant strains were located close to the Omicron variants, while others tended to spread toward other variants.

### Unsupervised pairwise data fusion using sparse CCA

**Fig. 3:**
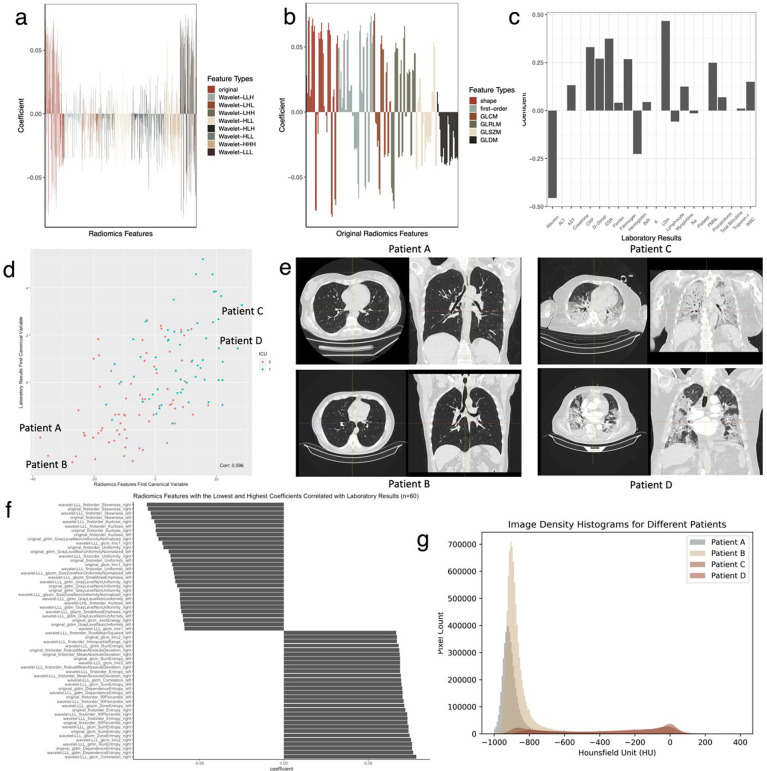
Sparse CCA Analysis of Radiomics Features and Laboratory Results. **a** Correlated radiomics features. Original and wavelet features in the LLL frequency channel have the highest absolute values of coefficients. **b** Coefficients of the original radiomics features. **c** Correlated laboratory results. Coefficients of laboratory results align with serum biomarkers related to severe disease and acute phase response. **d** The correlation between the first set of canonical variables shows that the first pair can capture the ICU outcome. We select two patients (Patient A and Patient B) with the lowest and two patients (Patient C and Patient D) with the highest canonical variables for radiomics features **e** Following the correlation plot, Patient A and Patient B’s CT images in axial and coronal planes have no pulmonary infiltration, whereas there are apparent findings on Patient C and Patient D’s CT images for COVID-19 pneumonia. **f** We select and visualize thirty variables with the highest and thirty variables with the lowest coefficients among the radiomics features. Histogram-based first-order features reporting the skewness, kurtosis, and uniformity have the lowest negative, whereas entropy-related features have the highest positive coefficients. **g** The image intensity histograms of the patients show that Patient A and Patient B have left-skewed histograms peaking around −1000 to −800 HU, consistent with air and lung parenchyma densities; however, histograms of Patient C and Patient D are flatter and more right-skewed, consistent with negative coefficients for skewness and kurtosis features and revealing a wider distribution of HU values.

Next, we performed sparse CCA analysis to examine the pairwise associations between all data modalities (Table 2). Relevant sparsity parameters corresponding to the highest Z-stat score were determined with the number of non-zero weights of X and Z. We first report the results for combining laboratory results and radiomics features for 127 patients $\left(corr\left(Xu_{1},Zv_{1}\right)=0.596\right.$, Figure 3). In the laboratory results group, LDH, which relates to disease progression and worse outcome, had the highest coefficient value (0.47), followed by erythrocyte sedimentation rate, D-dimer, polymorphonuclear leukocytes, white blood cell count, and acute phase reactants such as C-reactive protein and fibrinogen ^45^. At the same time, albumin had the lowest coefficient value (−0.46) as a negative acute phase reactant, along with hemoglobin, lymphocyte, and sodium levels. In the radiomics features group, original and wavelet features in the LLL frequency channel had the highest absolute values of coefficients. Among them, histogram-based first-order features reporting the skewness, kurtosis, and uniformity had the lowest negative coefficients, whereas entropy-related features had the highest positive coefficients. The negative coefficients of skewness and kurtosis features indicated that, as laboratory results worsened, since image density is measured approximately as −1,000 HU for air, and 0 to +70 HU for various tissue types such as blood, pleural effusion, abscess, and mucus, depending on the extent and content of the lung abnormalities, the image intensity histogram became flatter and more right-skewed ^46,47^. This led to a loss of homogeneity and increased entropy in the radiomics features (Figure 3).

**Fig. 4:**
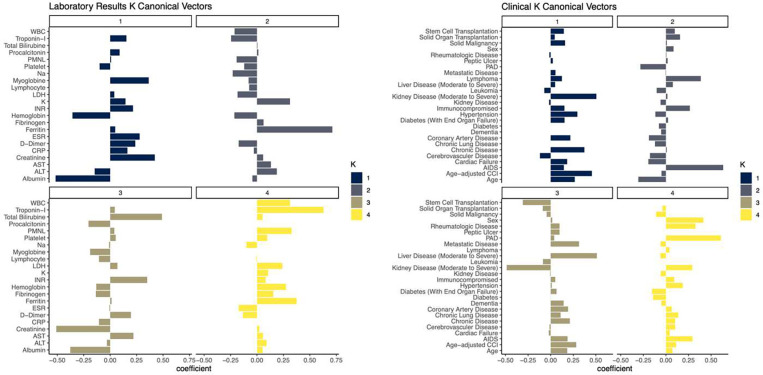
Sparce CCA Analysis of laboratory results and clinical data. The first four canonical variables are provided. Different canonical variables provide different clinical phenotypes: The first canonical variables represent a patient phenotype who is elderly, multi-morbid, and has moderate to severe renal disease with high creatinine and myoglobin levels, whereas the third canonical variables represent a different patient phenotype with moderate to severe liver disease with high bilirubin and INR levels.

Sparse CCA analysis of laboratory results and clinical data revealed different clinical phenotypes.$\left(corr\left(Xu_{1},Zv_{1}\right)=0.63\right.$, best L1 bound for X and Z: 0.7, p=0.04) (Figure 4). While laboratory results’ first canonical coefficients revealed a phenotype related to high creatinine levels with low albumin levels and anemia, on the clinical side, the patient appeared to be elderly and multi-morbid with moderate to severe renal disease. The second canonical variables represented a different patient phenotype who was young and immunocompromised with high ferritin levels. The third canonical variables also characterized a phenotype that likely presents a patient with a history of liver disease with elevated INR, total bilirubin levels, and hypoalbuminemia.

Next, we focused on the sparse CCA analysis of imaging and clinical data (Supplementary Figure 4). In this experiment, the permutation-based approach for choosing parameters provided a sparser solution than previous ones; the correlation coefficient was 0.65 (p<0.01). The first and third canonical vectors were found to be associated with sex, and the second and fourth ones were with lymphoma and CCI, respectively. Mainly_,_ the first sex-related canonical vector coefficients belonged to the left, and the second sex-related canonical vector coefficients belonged to the right lung and consisted of first-order and shape radiomics classes. In combinations of pairwise sparse CCA analysis of viral genome sequencing data with other data modalities, different encoding techniques for the viral genome altered the correlation plots, with better separation obtained with Viral-Word2Vec encoding (Supplementary Figure 5).

**Fig. 5:**
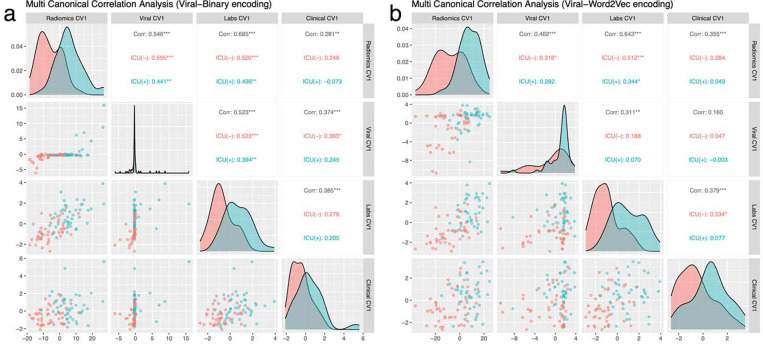
Sparse Multi-CCA Analysis of All Data Modalities: **a** The correlation pairs plot of the first canonical vectors of four data modalities, including Viral-Binary encoding. **b** Using Viral-Word2Vec encoding instead of Viral-Binary encoding provides a more homogenous distribution and better separation among canonical variables.

Finally, we performed sparse multi-CCA on patients with all data modalities (n=89). When Viral-Binary encoding was used for viral encoding, the highest Z-score was 2.15 (penalties = 12.22, 6.64, 1.56, 1.7, p<0.01). The highest Z-score was 3.14 (penalties = 17.923, 8.468, 2.293, 2.493, p<0.01) when Viral-Word2Vec was used for viral encoding. We showed that with the Viral-Word2Vec embedding, the viral genome projection of the patients was more homogeneously distributed, and pairwise correlation values of viral features and other data modalities were reduced (Figure 5). While the dominance of histogram-based first-order radiomics features and the importance of albumin and LDH among the laboratory results persisted in the analysis of these 89 patients, clinical data’s first canonical vectors slightly revealed a different clinical phenotype which was an elderly multimorbid patient with dementia (Supplementary Figure 6).

### Supervised multiview analysis using cooperative learning

We performed cooperative learning to build prediction models for the outcome of ICU hospitalization. First, we used all five data modalities to train unimodal prediction models in all patients. The highest score was achieved with the model using radiomics features (AUC=0.83 ± 0.01), followed by laboratory results and clinical data (AUC=0.77 ± 0.02 vs. 0.67 ± 0.02), respectively (Supplementary Table 2, Supplementary Figure 7).

**Fig. 6:**
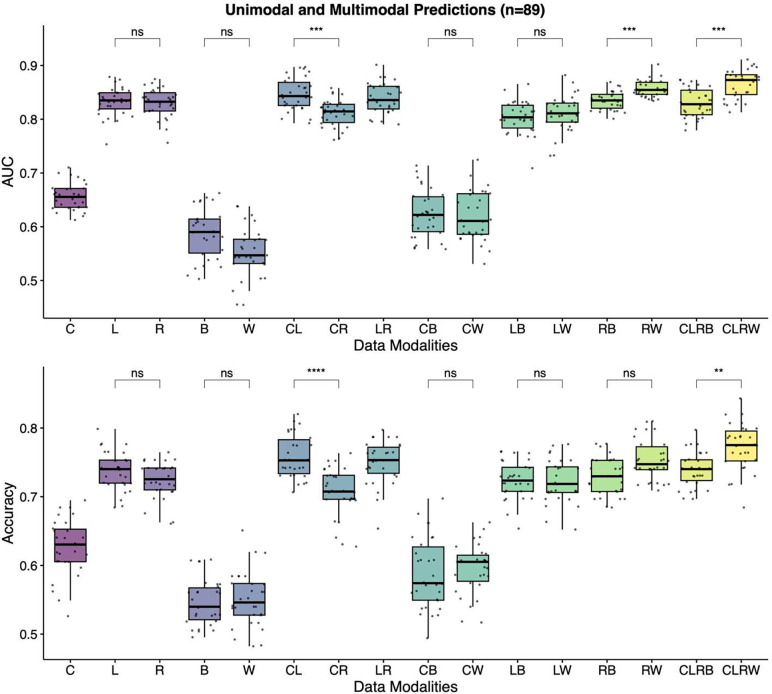
Unimodal and multimodal prediction models for the supervised task. The best accuracy and AUC values are achieved with the quadruple model using Word2Vec Encoding (CLRW). Abbreviations: C, Clinical data; L, Laboratory results; R, Radiomics; B, Viral-Binary encoding; W, Viral-Word2Vec encoding; AUC, area under the curve; ns, non-significant

Then, the models were trained with all single, double, and quadruple data combinations and evaluated for the same task in 89 patients. The best accuracy and AUC values were achieved with the quadruple model using Viral-Word2Vec encoding (CLRW) (Supplementary Table 3, Figure 6). While unimodal prediction models of radiomics features and laboratory results had a mean AUC score of 0.83, the quadruple prediction model using Viral-Word2Vec encoding achieved a mean AUC score of 0.87. The mean AUC score for the quadruple model using Viral-Binary encoding was 0.83 ± 0.2. There was a statistical difference between these two quadruple models (p<0.001). The model that combines radiomics and Viral-Word2Vec encoding also outperformed the model that combines radiomics and Viral-Binary encoding in terms of AUC scores (0.86 vs. 0.83, p<0.001). There was no statistical difference when we used different viral embedding techniques in other double combination models.

To understand which features were the most important for the performance of the CLRW model, we used all the available data as the training set. We performed a 5-fold CV to get the optimal hyperparameters and found Alpha and rho as 0.2 and 0.1, respectively. Model standardized coefficients were extracted at λ = 0.1 (Supplementary Figure 8). Again, like the results of the unsupervised sparse CCC analysis of radiomics and laboratory results, the original and wavelet features in the LLL frequency channel had the highest absolute values for the standardized coefficients. LDH, ESR, CRP, albumin, and total bilirubin were the selected serum biomarkers, and age, chronic disease, and CCI were the clinical variables of the CLRW model. Word2Vec encoding also contributed to the supervised task with its four dimensions.

## Discussion

The complexities of modern biomedical challenges demand more holistic approaches to data analysis. This study spotlights the potential of multi-modal data fusion, harnessing diverse data modalities to derive deeper insights into health phenomena. By integrating disparate data types, we can uncover nuanced patterns and relationships that single-modal analyses might miss. In particular, the potential of integrating viral genome sequencing, imaging, clinical data, and laboratory results is immense. Sparse CCA analysis and cooperative learning, as highlighted in our study, are instrumental in combining these data strands, offering a multi-faceted view of a complex disease such as COVID-19. Our exploration into using the Word2Vec NLP model for viral embedding further underscores the value of innovative techniques in transforming raw data into meaningful representations, especially in the context of viral genomic sequencing data.

Our approach has precedence. Using NLP techniques, particularly the Word2Vec model for viral embedding, has been recognized in prior research. But while earlier studies were often unimodal and focused on tasks like viral classification or evolution tracking, our method differs by integrating this with other data modalities, offering a more comprehensive view ^48–51^. The merit of this method is evident in its ability to encapsulate the cumulative effects of multiple viral mutations and their relationships, a task that single-modal approaches might find challenging. For example, we illustrated that Word2Vec encoding not only separates major SARS-CoV-2 variants but also allows the preservation of phylogenetic relationships among them. We also found that Word2Vec encoding is beneficial in showing which groups recombinant strains are close to, which might be challenging to represent in a phylogenetic tree.

Next, imaging data, particularly CT scans, holds potentially more information than can be observed by radiologists. Radiomics offers a quantitative approach to interpreting this data, allowing correlations with clinical features and laboratory results. Previous literature has highlighted the correlation between radiological findings and other biomarkers ^52–54^. Our analysis shows that serum biomarkers that represent positive and negative acute phase responses are mainly found to be correlated with radiomics features related to the distribution of voxel intensities. As known, in lung nodule and cancer studies, mainly shape-related features are robust and provide information about disease phenotypes and prognosis; however, our results emphasize the importance of histogram- and entropy-related features because a higher proportion of involved lung parenchyma, i.e., diffuse pulmonary infiltrates, is associated with severe disease ^55–57^. Furthermore, we reveal that biomarkers not playing a role in acute phase response, such as ALT, potassium, and creatinine, are not correlated with radiomics features as well.

Sparse CCA has been championed in various biomedical domains, from understanding eating disorders via imaging data to categorizing clinical subtypes in dementia ^58,59^. Our application to COVID-19 aligns with this trajectory, delineating clinical phenotypes like kidney disease, liver dysfunction, and age-related vulnerabilities, supported by existing literature and our clinical observations ^60–63^. However, challenges persist. Multi-modal data fusion demands rigorous computational techniques, nuanced strategies for feature extraction, and the transformation of raw data into a structured format. Our experiments show that when these elements align – as seen with the optimal results using Word2Vec encoding – the outcomes are promising. This performance edge over binary encoding likely arises because Word2Vec benefits from an expansive external database, capturing nuances not specific to our dataset. Decisions surrounding data fusion staging remain paramount. We employed cooperative learning, enhancing alignment across modalities, to determine optimal fusion, guided by the agreement penalty tuning. A review of the features selected in both our unsupervised and supervised models revealed a consistency, highlighting a harmony between an unsupervised use of sparse CCA analysis and supervised predictive cooperative learning.

A few caveats need to be noted. First, our relatively small sample size might be suffering from overfitting of data. To overcome this issue, we used a stratified nested CV framework for estimating the generalization performances of the trained models. Next, from a clinical point of view, this study is dated in the early days of the pandemic, and a small number of patients were vaccinated for COVID-19. Because it is not possible to quantify the vaccination effect properly, we had to ignore this effect.

To conclude, our findings reinforce the power and potential of multi-modal data fusion in biomedical research. Sparse CCA analysis and cooperative learning are pivotal tools in managing and interpreting high-dimensional data, as in the example of COVID-19. The Word2Vec model, as employed for viral genome encoding, is particularly promising, hinting at future directions for research in multi-modal biomedical data fusion.

## Figures and Tables

**Table 1:** Clinical characteristics of the patients

Variable	ICU (+) (*n*: 63)	ICU (−) (*n*: 86)	p-value

Age, years, mean (SD)	63.9 (15.0)	53.1 (15.6)	< 0.001

Female sex, *n* (%)	26 (41.3)	35 (40.7)	1

Comorbidities, *n* (%)			
Hypertension	38 (60.3)	34 (39.6)	0.02
Diabetes mellitus	22 (34.9)	22 (25.6)	0.29
Coronary artery disease	22 (34.9)	11 (12.8)	0.003
Solid organ malignancy	13 (20.6)	19 (22.1)	0.99
Kidney disease	13 (20.6)	10 (11.6)	0.20
Cardiac failure	14 (22.2)	6 (7.0)	0.01
Chronic lung disease	12 (19.0)	7(8.1)	0.08
Hematologic malignancy	4 (6.3)	7(8.1)	0.76
Rheumatologic disease	4 (6.3)	3 (3.5)	0.46
Cerebrovascular disease	2 (3.2)	3 (3.5)	1
Peripheric arterial disease	1 (1.6)	1 (1.2)	1
Dementia	2 (3.2)	0 (0.0)	0.18
Liver disease	0 (0.0)	2(2.3)	0.50

Immunocompromised, *n* (%)	12 (19.0)	16 (18.6)	1

COVID-19 vaccination history, *n* (%)	14 (22.2)	8(9.3)	0.05

Age-adjusted Charlson comorbidity index, median (IQR)	2 (1–3)	1 (0–1)	< 0.001

Steroid treatment (Minimum 6 mg/day dexamethasone), *n* (%)	51 (81.0)	23 (26.7)	< 0.001

Hospital LOS, days, median (IQR)	15 (10.5–31.5)	10 (6–14.75)	< 0.001

In-hospital mortality, *n* (%)	19 (30.2)	1 (1.2)	< 0.001

Abbreviations: ICU, Intensive care unit; LOS, Length of stay; IQR, Interquartile range

**Table 2:** Sparse CCA Analysis for Examining Pairwise Associations Between All Data Modalities. Sparsity parameters and correlations were calculated with the CCA.permute function. We chose the L1 bound for X and Z at the highest value of the Z-stat for each pairwise data modality.

Data Modalities		n	Best L1 bound for X and Z	Z-stat	p-value	Number of non-zero weights		Correlation
Radiomics	Lab Results	127	0.70 / 0.70	2.882	<0.01	1199	17	0.596
Radiomics	Clinical Data	127	0.10 / 0.10	3.423	<0.01	26	1	0.646
Radiomics	Viral-Binary E.	89	0.50 / 0.50	0.558	0.16	462	235	0.761
Radiomics	Viral-Word2Vec E.	89	0.70 / 0.70	0.584	0.20	775	241	0.524
Lab Results	Clinical Data	127	0.70 / 0.70	1.392	0.04	16	21	0.628
Lab Results	Viral-Binary E.	89	0.23 / 0.23	1.012	0.24	2	201	0.915
Lab Results	Viral-Word2Vec E.	89	0.10 / 0.10	0.489	0.20	1	6	0.576
Clinical Data	Viral-Binary E.	105	0.3 / 0.3	3.281	<0.01	17	328	0.982
Clinical Data	Viral-Word2Vec E.	105	0.7 / 0.7	0.214	0.36	20	206	0.487

Abbreviations: n, number; E., encoding

## Data Availability

The data supporting this study’s findings are available on request from the corresponding author. The data are not publicly available due to privacy or ethical restrictions.
